# You Talking to Me? Says the Enteric Nervous System (ENS) to the Microbe. How Intestinal Microbes Interact with the ENS

**DOI:** 10.3390/jcm9113705

**Published:** 2020-11-18

**Authors:** Mauro Giuffrè, Rita Moretti, Giuseppina Campisciano, Alexandre Barcelos Morais da Silveira, Vincenzo Maria Monda, Manola Comar, Stefano Di Bella, Roberta Maria Antonello, Roberto Luzzati, Lory Saveria Crocè

**Affiliations:** 1 Department of Medical, Surgical and Health Sciences, University of Trieste, 34149 Trieste, Italy; gff.mauro@gmail.com (M.G.); moretti@units.it (R.M); rma.roby@gmail.com (R.M.A.); roberto.luzzati@asugi.sanita.fvg.it (R.L.); lcroce@units.it (L.S.C.); 2 Italian Liver Foundation, 34129 Trieste, Italy; 3 Department of Advanced Microbiology Diagnosis and Translational Research, Institute for Maternal and Child Health-IRCCS “Burlo Garofolo”, 34137 Trieste, Italy; giusi.campisciano@burlo.trieste.it (G.C.); manola.comar@burlo.trieste.it (M.C.); 4 Neuroscience Laboratory, Human Anatomy Department, Biomedical Sciences Institute, Federal University of Uberlândia, 38408 Uberlândia, Brazil; alexandre.bm.dasilveira@gmail.com; 5 Diabetes Unit “Santissima Annunziata” Hospital, 44042 Cento, Ferrara, Italy; v.monda@ausl.fe.it

**Keywords:** enteric nervous system (ENS), microbiota, microbiome, gut, bacteria, parasites, viruses, gut–brain axis

## Abstract

Mammalian organisms form intimate interfaces with commensal and pathogenic gut microorganisms. Increasing evidence suggests a close interaction between gut microorganisms and the enteric nervous system (ENS), as the first interface to the central nervous system. Each microorganism can exert a different effect on the ENS, including phenotypical neuronal changes or the induction of chemical transmitters that interact with ENS neurons. Some pathogenic bacteria take advantage of the ENS to create a more suitable environment for their growth or to promote the effects of their toxins. In addition, some commensal bacteria can affect the central nervous system (CNS) by locally interacting with the ENS. From the current knowledge emerges an interesting field that may shape future concepts on the pathogen–host synergic interaction. The aim of this narrative review is to report the current findings regarding the inter-relationships between bacteria, viruses, and parasites and the ENS.

## 1. The Enteric Nervous System and the Gut–Brain Axis

The gastrointestinal tract has the function of digesting food, absorbing the nutrients, and forming a barrier against harmful agents, but it is also an immune-hormonal system. Functional aspects of this system, such as peristaltic movements, substance transport, and local blood flow, are regulated by an intrinsic network of neuronal ganglia known as the enteric nervous system (ENS) [1]. The ENS provides motor excitatory neurons, innervate muscle layers, secretory glands, and the lymphatic vascular system. It is the largest and most complex part of the peripheral nervous system, being organized into distinct neuron networks within the gut wall, where individual small ganglia are interconnected by dense fiber bundles. These nerve plexuses are organized into two layers of neuronal ganglia and enteroglial cells that are interconnected: The myenteric plexus (Auerbach plexus) and the submucosa plexus (Meissner plexus). The ENS forms a complete sensory-motor reflex composed of intrinsic primary afferent neurons (IPANs), interneurons, and motor neurons [2].

These plexuses are located between the layers of the gastrointestinal tract and present about 20 subtypes of neurons, differentiated by the expression of neuropeptides [1]. The ENS also features enteroglial cells (EGCs). These are relatively small, with a star shape and can be identified immunochemically through the expression of specific proteins, such as glial fibrillar acid protein (GFAP), vimentin, and S-100 [3]. These cells can express receptors for cytokines, neuropeptides, and neurotrophins, exerting functions in both the ENS and the immune system, and participating in the modulation of motility and secretion functions of the gastrointestinal tract. In addition, EGCs are necessary for the structural and functional integrity of the ENS, participating in the intestinal mucosal barrier and contributing to intestinal homeostasis [4].

In 2013, the National Institute of Mental Health launched a project focused on exploring the mechanism involved in gut–brain communication [5]; however, the exact mechanisms by which the gut and brain communicate and influence each other are not yet fully understood. From an anatomical point of view, the critical interactive communications between the gut and brain are the sympathetic system (SS) and the vagus nervus (VN) of the autonomic nervous system (ANS), while the site of interactive communication occurs in the spinal cord [5]; a sophisticated and four-leveled control system has been well-described [6,7]. The very first level occurs in the ENS, where it depends on the myenteric ganglia, sub-mucosae ganglion, and EGCs [8,9]. Proceeding in an ascending pathway, the second control step occurs in the prevertebral ganglia, which mediates visceral reflex responses [10]. The third hierarchical level is located in the spinal tract between T5 and L2, for the SS, and S2 and S4/S5 for the parasympathetic system, through the tractus solitaires nucleus in the brain stem and the dorsal motor nucleus of VN, whose effect is dominant in the upper gastrointestinal tract, mediated by cholinergic inputs [11]. Cortical and basal ganglia neurons maintain the highest level of control. The afferent fibers of the VN arrive at the nucleus of the tractus solitaries, whose fibers ascend towards the thalamus. Spinal afferent fibers ascend within the spinothalamic tract, enter the gracile nucleus and cuneate nucleus, and project to the thalamus through the lemniscus medialis. These fibers arrive diffusively in the lobus limbicus, which is the insular cortex, through the parabrachialis nucleus [5,12]. The VN represents the primary neural pathway connecting the gastrointestinal tract to the solitary tract’s nucleus and from that to the hypothalamus and neocortex [13]. The VN does not directly interact with the gut luminal content [14], despite being indirectly related through metabolites altered by the entero-endocrine cells in the gut epithelium [14]. This system has recently been described, with a complicated relationship between vagal synaptic afferents and entero-endocrine cells in the gut, which probably directs nutritional information towards the brain, mediated by the glutamatergic neurotransmission [15]. VN fibers are enriched with receptors such as 5-HT3, Toll-like receptor 4 (TLR4), and free fatty acid receptors (FFARs), and their final projections end in the brain [14].

In the gastrointestinal tract, a wide variety of neurotransmitters, neuro-regulators, and hormones play different roles. Acetylcholine (ACh) acts via muscarinic receptors to directly stimulate smooth intestinal muscle contractility [16]. Substance P (SP), neurokinin A, and neurokinin B are neuromodulators of tachykinin, and the action of SP on neurotransmission occurs in the non-adrenergic/non-cholinergic system (NANC), which is directly involved in the perception of painful stimuli. The vasoactive intestinal peptide (VIP) induces vasodilation and modulates mucin secretion and the proliferation of goblet cells in the intestinal mucosa [17]. In addition, it participates in the relaxation of intestinal smooth muscles and modulates functions of the lymphocyte component of the immune system. Cholecystocin (CCK) is a major mediator of gastrointestinal feedback to the central nervous system through the afferent component of the VN. Histamine and serotonin (5-hydroxytryptamine or, simply, 5-HT) modulate the function of a variety of intestinal cells, including neurons, EGCs, muscle cells, and the immune system. Somatostatin (SST), which lies behind the regulation of the growth of intestinal cells, inhibits the secretion of gastrin, insulin, glucagon, and cytokines [18,19].

The aim of this narrative review is to report the current findings regarding the inter-relationships between bacteria, viruses, and parasites and the ENS.

### Circuitry of the Enteric Nervous System

The neurons of the ENS communicate with each other using the same “language” as in the central nervous system. From a functional point of view, reflexes in the ENS can be divided into two major categories: (1) Axon reflexes, where a thin afferent nerve fiber is connected to the central nervous system (often activated by noxious mucosal stimuli), with the afferent fibers making contact with an effector cell (i.e., epithelium, blood vessels, or another neuron), and (2) intramural reflexes, i.e., reflexes confined to neurons contained within the gastrointestinal wall [20]. In the latter, a noxious intraluminal stimulus (e.g., bacterial toxins) can activate the endocrine mucosal cells to produce peptides, which reach the nervous terminals of the submucosa plexus, in turn stimulating the myenteric plexus, which exerts its action on post-ganglionic fibers and the final effectors (e.g., epithelial cells, vessels, and muscles) [21].

## 2. Commensal Bacteria and the Enteric Nervous System

The human gastrointestinal system is inhabited by a large group of 1000 distinct species of bacteria in a symbiotic relationship [22]. This variegate collection of microbes is called the “microbiota”, whereas their genetic material is referred to as the “microbiome” [22]. The commensal microbiota colonizes the mammalian gut and other body surfaces shortly after birth and remains there throughout an individual’s lifetime. In healthy adult individuals, the microbiota is primarily composed of five bacteria phyla: Firmicutes (79.4%); Bacteroidetes (16.9%); Actinobacteria (2.5%); Proteobacteria (1%); and Verrucomicrobia (0.1%) [23]. Although bacteria are the most represented biological entities, fungi, archaea, and viruses create the “rare biosphere” of the gut microbiome. A healthy and balanced state marked by a high diversity and abundance of microbial populations in the gut is defined as *eubiosis* [22]. A wide range of factors, including diets with highly processed foods, a lack of regular sleep, and several diseases, can alter the microbiota diversity and abundancy (*dysbiosis*) [22]. The dysbiotic state has been linked to several pathological conditions, such as cardiovascular disease, obesity, diabetes, inflammatory bowel disease, and pulmonary hypertension [22,24].

It has long been thought that the only control exerted by the gut microbiota occurs through the VN and ENS [25,26]. Surprisingly, it has been demonstrated that, even though VN and ENS are fundamental in the gut–brain axis, the microbiota plays a critical role in immune, endocrine, and neuroendocrine maturation in nervous system sprouting [25]. It is interesting to note that all of the actors (VN, ENS, and microbiota) are co-primary in their contribution to brain afferents. For example, the capacity of the bacterium *Lactobacillus rhamnosus* JB-1 to modulate anxiety-like behavior and gamma-aminobutyric acid (GABA)-mediated neurotransmission in mice is lost after vagotomy, and the anxiolytic effect produced by *Bifidobacterium longum* NCC3001, which is absent in mice after vagotomy, suggests a fundamental role of the VN and ENS in the modulation of bacteria [27,28]. On the other hand, a mild gastrointestinal infection, after vagotomy, generates anxiety, with a presumed direct effect in the brain [29].

The gut microbiota synthesizes different metabolites, i.e., esters, serotonin, tryptophan, and various fatty acids, which might influence the brain. It has been demonstrated that the indirect effect exerted by the gut microbiota influences serotoninergic transmission, regulating tryptophan, whose concentration is higher in male germ-free mice compared to controls with an intestinal microbiota [30], who also show a higher hippocampal concentration of serotonin [14].

**Figure 1:**
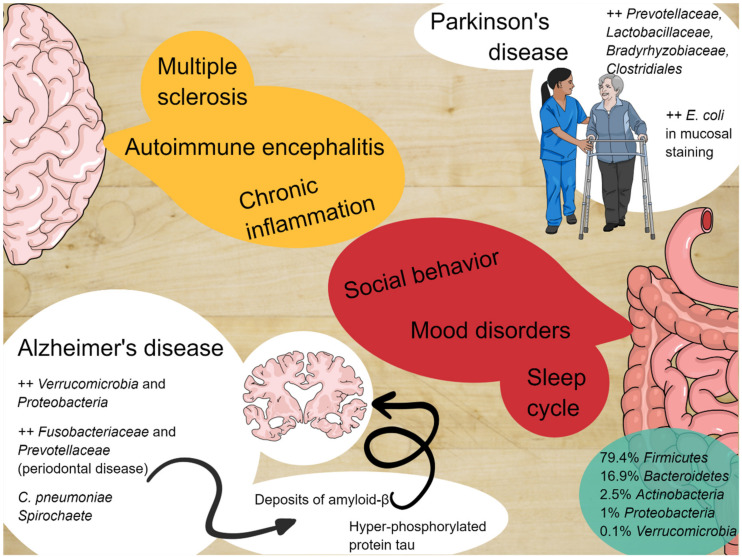
The enteric nervous system and the central nervous system constantly communicate with each other and alterations of the microbiota can be involved in the pathogenesis of several diseases.

It has been well-demonstrated that the microbiota produces short-chain fatty acid (SCFA) metabolites, i.e., butyrate, propionate, and acetate. They have a direct effect on repairing microglia in germ-free mice [31,32]. Furthermore, SCFAs impact at least two systems of molecular signaling that have widespread regulatory effects throughout the body: Histone deacetylation (HDAC) and G-protein-coupled receptors (GPCRs) [33]. SCFAs are natural inhibitors of HDAC and activators of specific G-protein-coupled receptors (GPCRs). An imbalance in the direction of excessive HDAC has been found in Parkinson’s disease [34]. GPCRs are transmembrane proteins that represent a significant gateway through which cells convert external cues into intracellular signals (29). SCFAs activate two specific GPCRs (GPR41 and GPR43) with no other known ligands [35,36]. GPR41 is abundant in human sympathetic ganglia, where its activation by propionic acid increases sympathetic nervous system outflow and one potential mechanism by which dietary fiber (rich in SCFA) can increase the basal metabolic rate and help control obesity [36,37]. It has also been proven that propionate and butyrate administered to rat neuroblasts increased the expression of tyrosine hydroxylase, which is the rate-limiting enzyme in noradrenaline, and dopamine synthesis [14,38]. The main findings in terms of the bacterial influence on the ENS are reported in Table 1 and represented graphically in Figure 1.

### 2.1. Microbiota and Social Behavior

Different studies have remarked on the fundamental role of the gut microbiota in social behavior. This probably involves the horizontal transmission of microbes between conspecifics [14], for example, in specific Blattodea, through social events such as coprophagia and proctodeal trophallaxis or in social bees [39]. The presence of *Bifidobacterium* and *Lactobacillus* in their gut is fundamental for SCFA production, which becomes essential for nutrition in starvation periods [40,41]. Different social behavior, such as grooming, in Baboons, is determined by the convergence of core gut microbial taxa [42]. The data seem even more impressive when considered that mice born from mothers on a high-fat diet present an altered microbiota composition, with a significant reduction of *Lactobacillus* spp. and a reduced ability to discriminate between familiar and unknown conspecifics [43]. This defect can be replaced by *Lactobacillus reuteri*, with a consequent increment of oxytocin, in the paraventricular nucleus of the hypothalamus [44], improving their social conduct [14].

Alterations of the gut microbiota, associated with a lack of expression of Toll-like receptors (TLRs), contribute to the altered response of different pathogens in the gut, i.e., a TLR4-knockout mouse does not show any response to lipopolysaccharide (LPS) produced by gram-negative bacteria [45,46]. The Griseofulvin Mouse model, compared with a specific-pathogen-free mouse model, produced significantly elevated corticosterone and adrenocorticotropic hormone levels when exposed to stressful conditions. This production could be partially reversed by a fecal microbial transplant, and was ultimately reversed over time by single *Bifidobacterium infantis* [47]. Moreover, the experimental conditions reveal that, on that occasion, the timing of the microbiota modeling answer is very limited in time-span, being fundamental for a precocious maturation of the hypothalamus–pituitary–adrenal axis, with a gender-specific response [31,48]. More recently, many studies have documented that the microbiota of long-term stressed mice was significantly different from that of a non-stressed mouse [49]. One study also showed that prolonged stress reduces the quantity of *Bacteroides* at the cecum and increases the amount of *Clostridium* [49]. Namely, three kinds of stress-induced bacteria of *Enterococcus faecalis*, *Pseudobutyrivibrio*, and aerogenic bacteria of the *Dorea* strain have been found [5,49].

Experimental models of germ-free and antibiotic-treated animals, both of which determine a total absence of microbiota, show macroscopic alterations of neurotransmitter turnover, an altered neuronal morphology, and significant neuroinflammation [49], depending on the time of microbiota onset. Likewise, a substitution of the microbiota results in a drastic modification of behavior and social conduct in experimental animals, such as rodents. On the contrary, supplementation with *Bifidobacterium* and *Lactobacillus* can lead to notable improvements in social behavior in early life and adulthood [14]. As admirably written by Sherwin et al. [14], “Emerging research is now conceptualizing animals as “holobionts”: dynamic ecosystems, comprising a host and its associated microorganisms, that can vary with time, localization, and function. Collectively, the host and microbial genomes of a holobiont are termed a hologenome, and variations in the hologenome caused by changes in the host and/or microbes may affect phenotypes may be subject to natural selection”.

### 2.2. Microbiota, Sleep Cycle, and Mood Disorders

According to Sherwin et al. [14] and many others [44,50,51,52,53,54,55,56,57,58,59,60], it seems quite essential that the microbiota persistently stimulates the immune system, but this remodeling effect has a consequence, even if very distant [37,59]. A possible interface between the gut microbiota and sleep regulation has been suggested. It has been widely described that the gut microbiome produces and activates intestinal macrophages, inducing the production of IL-1β and TNF-α [60]; inside the intestinal wall, many LPS induce the synthesis of IL2-IL18 [61,62,63]. The intimate relationship between TNF-α, IL-18, and NREM sleep has been described [62,63]. It is also well-accepted that cortisol inhibits the synthesis of these cytokines in the gut-microbiota, and IL-1β and TNF-α display a peak level in human blood around midnight, when cortisol is at the nadir [64,65]. The parenteral administration of LPS to humans in nanogram quantities (0.4 ng/kg body weight) increases the plasma concentration of IL-6 and TNF-α, along with salivary and plasma cortisol and plasma norepinephrine. These changes are accompanied by a depressed mood, increased anxiety, and impaired long-term memory for emotional stimuli [66,67]. Matsuda et al. recently developed a depression rat model using the 14-day social defeat stress (SDS) paradigm [68]. These experimental rodents exhibit long-term social avoidance, major depressive disorders, and sleep abnormalities, with increased REM, but a decreased NREM sleep time and increased defragmentation of sleep continuity. The authors examined the fecal gut microbiota before, during, and after stress studies. The social defeat stress significantly increased the fecal classes of Betaproteobacteria and Flavibacteria, while decreasing those of Clostridia. *Bacteroides* and Bacilli showed a tendency to increase, whereas Actinobacteria tended to decrease. When compared to before stress, *Lactobacillus* showed evident decreases, whereas *Blautia* exhibited significant increases. The *Lactobacillus reuteri* levels significantly increased following stress conditions, with further increases observed even being observed one month after the stress conditions ended [68]. Conversely, other species (*Ruminococcus flavefaciens*, *Blautia producta*, and *Clostridium perfringens*) exhibited only temporary change [67,68].

It has been demonstrated that an altered gut-microbiome with elevated LPS and peptidoglycan is regularly higher than that of a teetotaler; in alcoholics, before and during ethanol detoxification, there is an increased mRNA and plasma level of IL-8, IL-1β, and IL-18. Employing Cr51-EDTA as a probe of intestinal permeability, a population of chronic alcoholics was studied, who were divided into two groups: Those with high and those with normal permeability (65). The high permeability group had higher scores of depression, anxiety, and alcohol craving than the low permeability group, as well as a distinct pattern of changes in the gut microbial population, characterized by decreased colonization of bacteria known to have anti-inflammatory effects; *Bifidobacterium* species; and *Faecalibacterium*, in particular, *Faecalibacterium prausnitzii* [37,69]. Alcoholics who displayed the persistence of intestinal hyper-permeability after three weeks of ethanol withdrawal also demonstrated the persistence of depression, anxiety, and alcohol craving [69].

### 2.3. Microbiota and Alzheimer’s Disease

Alzheimer’s disease (AD) is a chronic and irreversible neurodegenerative disease, characterized by a loss of neurons and progressive impairments in the synaptic function, accompanied by deposition of the amyloid-β (Aβ) peptide outside or around neurons, together with an accumulation of hyper-phosphorylated protein tau inside cortical neurons [70,71,72,73,74]. Amyloid accumulation, involving the deposition of hyperphosphorylated tau proteins, with consequent microtubule destabilization, leads to two critical processes: An essential increase of general neuro-inflammation, and significant microglial and astrocytic activation and starvation of the neurons, due to the interruption of axonal transport. The most important consequences are the altered glutamatergic currents and the critical calcium inflow currents, with the significant induction of apoptosis [73,75,76,77]. Animal models of amyloid and tau depositions are related to herpes simplex virus type 1 (HSV1) infection in mice that upregulates the encoding genes for cholesterol 25-hydroxylase (CH25H), which seems to be involved in amyloid altered catabolism or hyper-production [78,79,80]. Nevertheless, many other bacteria have been related to the essential neuro-inflammatory status, typical of AD, such as spirochaete, and *Chlamydia pneumoniae* [78,81,82]. A positive relationship with phosphorylated tau and phosphorylated tau/Abeta 42 in cerebrospinal fluid and microbiota metabolism has been found with an elevation of trimethylamine N-oxide in AD models [83]. Moreover, transgenic wild-type amyloid precursor protein (APP) mice and germ-free mice have a diminished level of amyloid deposition compared to APP mice with a healthy microbiota [84], and this has also been proved in long-term spectrum antibiotic treatment, which seems to reduce amyloid depositions [85]. It has been described that AD mice have severe quotes of Verrucomicrobia and Proteobacteria, with a concomitant reduction of *Ruminococcus* and *Butyricicoccus* and short-chain fatty acids [86,87]. Moreover, poor oral hygiene has been linked to AD, with parodontopathy and tooth loss being risk factors for dementia in two studies [88,89], as well as severe periodontitis related to lower cognitive functions [90,91,92,93,94,95,96,97]. Even if studies have many different biases, it has been demonstrated that periodontal disease may be related to an increased brain amyloid load through PET studies [93], and that there is an increase of Fusobacteriaceae and higher abundance of Prevotellaceae in AD patients [93,98].

### 2.4. Microbiota and Parkinson’s Disease

More studies have been conducted on the second most frequent form of neurodegenerative disease, which is Parkinson’s Disease (PD). The converging line of these studies shares two common points: The high-density microbic population of the olfactory bulb and the gut, and the high-density deposition of misfolded alpha-synuclein deposition at the two sites [12,99,100,101]. It has been demonstrated that the alpha-synuclein deposits have a rostrocaudal gradient [102], with a higher concentration in the submandibular gland and lower concentration in the esophagus [102,103]. It has been suggested that the main vagal efferents could be the sprouting routes from peripheral sites towards the brainstem [100], and a vagotomy decreased the adjusted risk of developing PD in a 20-year-followed-up population [104,105]. It has been demonstrated that there is a higher intestinal permeability in PD patients [106], with a higher presence of Enterobacterales (*E. coli*) in mucosal staining, associated with a higher plasmatic LPS binding protein in PD patients [106,107]. In wild-type over-expressed alpha-synuclein mice (ASO), germ-free conditions produce fewer motor symptoms and minimal signs of general brain inflammation and alpha-synuclein [108]. The same aspects occur in antibiotic-treated mice, whereas colonization with wild--type mice or healthy subjects feces, or with high quantities of SCFAs, determines a worsening of Parkinsonian motor symptoms [106]. Three cross-sectional studies reported a relative abundance of Prevotellaceae in PD, but not in controls [109]. Combined with the severity of constipation, the abundance of Prevotellaceae, Lactobacillaceae, Bradyrhyizobiaceae, and Clostridiales IV could be used to identify PD cases with a 66.7% sensitivity and 90.3% specificity. Postural instability and gait symptoms were associated with the relative abundance of Enterobacterales [102,109,110]. It has also been reported that there is an increment of LPS synthesis in PD subjects relative to controls [110]. The same aspect has been reinforced in a different study, which showed lower serum levels of LPS-binding protein [107], as well as a reduction of the absolute concentration of fecal SCFAs (acetate, propionate, and butyrate) [111].

### 2.5. Microbiota and Other Neurodegenerative Disease

Several studies in animal and human models of demyelinating diseases, i.e., experimental autoimmune encephalomyelitis (EAE), Multiple Sclerosis (MS), and Devic’s Neuromyelithis (NMO), have been performed [112,113,114,115,116].

Germ-free mice were highly resistant to developing autoimmune encephalitis [113,114] or had lower clinical scores due to their encephalitis [114]. However, this condition was acutely reversed when these germ-free mice received a fecal transplant from healthy mice [116]. It has been thought that environmental conditions which seem to influence MS progression, such as obesity, smoking, low vitamin D levels, and altered responses to human viruses [23,117,118,119,120], seem to do so through the mediation of microbiota [121,122]. Leaky gut [123] is highly present in relapsing-remitting MS, and different studies have shown a different gut microbiota composition in MS, rather than in control subjects [123,124,125,126,127,128]. Any specificity has been remarked on for a given microbiota composition in MS cases, but a pro-inflammatory milieu is a constant finding [102,123]. Overlap with other inflammatory chronic conditions, such as Crohn’s disease, small intestinal bacterial overgrowth, rheumatoid arthritis, and undifferentiated connective pathologies, has been documented [129,130,131]. There is a tendency to report some specific groups of microbes in MS microbiota, i.e., Archaea (genus *Methanobrevibacter*) [125], or the depletion of Firmicutes species (*Clostridium* genera) [123] and Bacteroidetes phyla [126,127,128]. Even animal models of EAE (primates) might show low levels of Lipid 654, which is a lipopeptide, presumably derived from gut Bacteroidetes [132]. Minimal studies have been done to determine MS levels and disease-progression, and a definite result could be obtained through such investigations. Nevertheless, in a pediatric MS population, the depletion of Fusobacteria was associated with a higher hazard ratio of an earlier relapse [123], and different studies are currently being conducted on this fascinating topic [133].

The scenario for NMO is different, which is frequently associated with anti-aquaporin4 and the presence of the *Clostridium* adenosine triphosphate-binding cassette transporter in the gut microbiota [134,135], even if all recruited patients with NMO undergo Rituximab therapy [135,136].

## 3. Pathogenic Bacteria and the Enteric Nervous System

In addition to commensal microbiota, pathogenic bacteria in the lumen also interact with the ENS indirectly though non-neuronal intermediary cells, such as endocrine (in particular, enterochromaffin cells (ECs)) or immune resident gut cells. Some enterotoxins evoke intestinal secretion via nervous reflexes, occurring in parallel to toxins and having a direct secretive effect on intestinal cells.

In addition, a local gut infection can lead to subtle changes in the affective state and emotional responsiveness, as in the case of *Campylobacter jejuni* rodent models, which developed anxiety-like behavior in the absence of a systemic immune response. In particular, *Campylobacter jejuni* infection was associated with an elevated expression of the neuronal activation marker c-Fos in neurons located in the vagal sensory ganglia and in the nucleus of the solitary tract, as well as in brain regions associated with primary viscerosensory pathways and the central autonomic network [137].

### 3.1. Toxins Promoting Secretion

Among toxin-producing bacteria, *Vibrio cholerae* produces a potent exotoxin which causes hypersecretion in the small intestine. The cholera toxin (CT) consists of an A (or enzymatic) subunit of 28 kDa, and five B (or binding) subunits of 11 kDa each, assembled in a pentameric molecule. The implication of ENS in the pathophysiology of cholera infection was initially proposed in 1980 [138]. Previous evidence suggested that CT activated persistent cAMP-dependent release of 5-HT from the mucosa, resulting in activation of the secretomotor reflex pathways (via the 5-HT_3_ receptor) in the ENS by the activation of dendrites of submucosal plexus neurons, and eventually resulting in the release of VIP and its binding to enterocytic receptors, thus activating further cAMP production, promoting water and electrolyte secretion [20,139]. However, recent evidence suggests that CT increases the excitability of neurons of the myenteric, but not submucosal, plexus, and that neurokinin 3-receptors and not 5-HT_3_ receptors are involved in the neurosecretory reflex [140]. The heat labile enterotoxin (HLT) produced by *E. coli* shares a structural homology of 88% with the CT and induces a much less severe form of diarrhea [141]. Moreover, HLT inoculation does not stimulate the release of 5-HT from ECs. The HLT mechanism appears to be intricately related to the ENS, given that the administration of ganglionic blocked had preventive effects on the development of diarrhea [141]. Furthermore, the much smaller *E. coli* heat-stable enterotoxins (STa) of 2-5 kDa seem to activate an NO-dependent myenteric plexus secretory reflex mediated by capsaicin-sensitive C fibers [142,143], in addition to response suppression by VIP antagonists. In this case, VIP and NO appear to have a synergetic effect, since NO promotes VIP secretion from nerve terminals. In addition, STa can activate neurokinin receptor 2, which can further promote intestinal secretion [144].

*Clostridioides difficile*, which is another toxigenic bacterium, is the primary cause of antibiotic-associated diarrhea and colitis in humans. Toxigenic strains release two exotoxins: Toxin A (TX-A) and (TX-B). These are responsible for diarrhea and an acute mucosal inflammatory response [145]. The introduction of *C. difficile* toxins into the gut lumen stimulated the influx of neutrophils and promoted the activation of enteric neurons to increase luminal secretion and peristalsis [146]. Additionally, responses to TX-A involve the up-regulation of substance P in both lumbar dorsal root ganglia and small bowel enterocytes [147]. In addition, in vivo models showed that low doses of TX-A solicited an excitatory action at the level of the submucosal plexus and were involved in the suppression of noradrenaline release from sympathetic postganglionic axons [148]. As a result, the stimulation of submucosal secretomotor neurons evokes secretion from mucosal crypts. The inactivation of sympathetic braking on secretomotor neurons further facilitates the secretion. Moreover, recent studies showed that EGCs are susceptible to *C. difficile* infection, due to the cytotoxic and senescence-promoting effects of TX-B [149,150].

### 3.2. Toxins Promoting Emesis

*Staphylococcus aureus* produces a myriad of enterotoxins (SEs), commonly responsible for food poisoning. It appears that emesis is caused by 5-HT secretion. In particular, it may be related to 5-HT3 receptors located in VN sensory terminals that project up to the emetic center in the brainstem [151]. However, the process has only been proven indirectly in animal models of *Suncus murinus*, given that emesis is prevented by 5-HT inhibitor and 5-HT3 receptor antagonists [151]. A similar mechanism has been proposed for cereulide, which is a cyclic dodecadepsipeptide that is produced by *Bacillus cereus*. The emetic effects of the toxin seem to be dependent on 5-HT3 receptors on VN afferent neurons since vagotomy and 5-HT3 receptor antagonists inhibit emesis in *Suncus* [152]. Similar to SEs, it is not known whether cereulide directly interacts with VN sensory endings or promotes 5-HT release by ECs [152].

## 4. Viral Influence on the Enteric Nervous System

**Figure 2:**
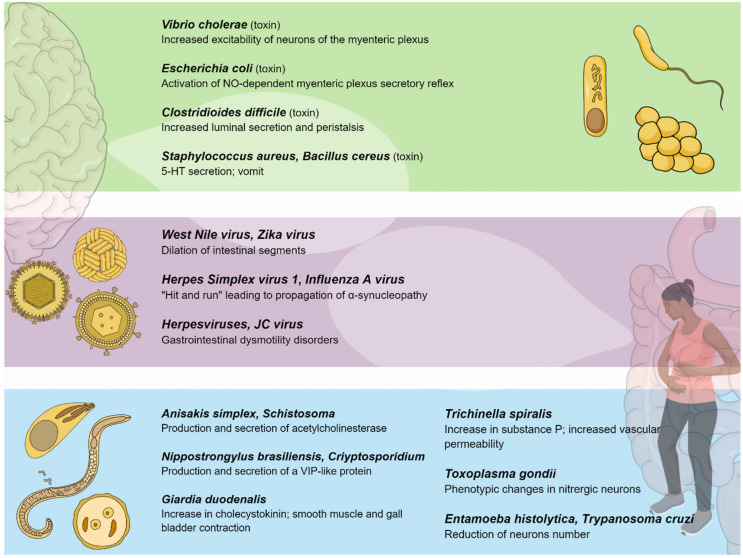
Pathogenic bacteria, viruses, and parasites primarily affecting the gastrointestinal system interact with the enteric nervous system, establishing a connection between the gut and the central nervous system.

Several gastrointestinal motility disorders (GIMDs) can depend on functional or anatomic alterations of the ENS [153,154,155]. The molecular basis of these alterations is heterogeneous, including degenerative and inflammation-mediated abnormalities [156]. In this context, infectious agents, such as neurotropic viruses, can be identified as etiological factors affecting the integrity of the ENS, either directly or through immune-mediated mechanisms [157]. The main findings in terms of the viral influence on the ENS are reported in Table 2 and represented graphically in Figure 2.

Evidence supporting viruses as possible etiological factors involved in GIMDs is still based on sporadic cases or small case series reporting the occurrence of virosis (e.g., poliomyelitis, influenza, and herpes zoster) before the onset of achalasia [158] and neurogenic intestinal pseudo-obstruction [159,160]. Basically, neurotropic viruses associated with GIMDs are likely to enter the ENS through the gastrointestinal mucosa and possibly establish latency with cyclic reactivation [161].

Viruses can target different cellular populations of the ENS. The EGCs are the major component of the ENS that can be targeted by viruses and outnumber enteric neurons by a factor of 4 to 10 [162]. EGCs act as a mechanical support for enteric neurons, are responsible for the survival and differentiation of neurons [163], and are antigen-presenting cells to innate and adaptive immune cells [164,165]. Then, EGC activation by viruses or their antigens is a key step for peripheral neuroglial immune priming by viruses, leading to a late onset of neurological impairment [166]. Inflammatory stimuli activate EGCs and convert them into a “reactive glial cell phenotype” which can release protective factors (neurotrophin-3, GDNF, GNSO, and PEA/PPAR-α) or destructive factors. The massive release of destructive factors and several proinflammatory mediators, such as IL-1β, IL-6, TNF-α, and MCP-1, alters the gastrointestinal motility [167,168,169].

Some cases of intestinal dysmotility disorders seem to be related to viral infection [170,171,172]. In mice models, inoculation with neurotropic flaviviruses leads to the injury and death of enteric neurons, inflammation, intestinal dilation, and retarded bowel transit. More precisely, the inoculation of mice with the flaviviruses West Nile virus (WNV) and Zika virus (ZIKV) leads to viral replication throughout the intestinal tract and the dilation of intestinal segments. Viral replication has been specifically observed within enteric neurons, causing cell death, but not in glial cells or mucosal epithelial cells. In addition, animals surviving WNV infection show defects in gastrointestinal motility from 4 to 7 weeks after infection. Overall, these observations support the hypothesis that some gastrointestinal dysmotility disorders may be episodic following infection/inflammation or chronic, which can be periodically exacerbated by additional infections or inflammation [173].

A delayed effect of viral infection can be hypothesized in the initial neuropathogenesis of Parkinson’s Disease, according to the “viremic hit” hypothesis, which is based on a dual-hit theory. In particular, influenza A virus may “hit and run”, initiating pathological alterations in the ENS structures, whereas HSV-1 may “hit” and establish life-long persistency with repetitive reactivations from latency, depending on the level of immunosenescence [174]. These viremic hits might induce the formation of α-synuclein fibrils in the peripheral nervous tissues, leading to the gradual transneuronal propagation of α-synucleopathy within the brain [175,176,177].

Additional neurotropic and gastrointestinal tract-infecting viruses should be considered as good candidates for causing human gastrointestinal dysmotility disorders. In this regard, some cases of chronic intestinal idiopathic pseudo-obstruction (CIIPO) [178] have been associated with viral infections in both pediatric [179,180] and adult patients [159,181,182]. Among the candidate infectious agents, herpes family virus, varicella zoster virus (VZV), cytomegalovirus (CMV), Epstein–Barr virus (EBV), and JC virus infections have been identified. Basically, it is believed that viral infections can affect the neuromuscular layer of the gut. In particular, VZV may infect and establish latency in ganglia of the ENS [157] and it has been associated with acute colonic pseudo-obstruction (Ogilvie’s syndrome), severe abdominal pain preceding fatal varicella, autonomic dysfunction, and intestinal pseudobstruction symptoms following glandular fever secondary to EBV infection [180]. In comparison, EBV has been associated with myenteric ganglionitis, characterized by inflammatory infiltrates within the myenteric plexuses [180]. JC virus has been identified in the enteroglial cells of the myenteric plexus in some patients with CIIPO [183].

EGCs have also been identified as an HIV target. The viral HIV-1 Trans activating factor (HIV-1 Tat) protein is hypothesized to be responsible for diarrhea and neurotoxic effects. One hypothesized mechanism is that HIV-1 activates glial cells, causing a neuroinflammatory response, which can be propagated to the central nervous system. Specifically, HIV interferes with the nervous system function by infecting EGCs, which release HIV-1 Tat, inducing an alteration in enteric neurons’ action potential by increasing Na+ channel expression [166,184]. In addition, Tat can interact synergistically with morphine, being able to activate EGCs and worsen GI dysfunction in HIV-infected narcotic users and HIV-infected patients, using opioid drugs to treat diarrhea [185,186]. Other interactive pathways have been shown between HIV-1 Tat protein and LPS. In mice models expressing Tat, bacterial intestinal translocation is significantly increased. Consequently, Tat and LPS synergize to induce the release of the pro-inflammatory cytokines IL-6, IL-1β, and TNF-α. More specifically, HIV-1 Tat is able to interact with the TLR4 receptor to enhance the pro-inflammatory effects of LPS [187].

EGCs can be activated by ECs infected with viruses. ECs are distributed along the intestinal mucosa to release mediators from the basolateral surface and to activate afferent neuron endings, mainly within the lamina propria [188]. Among their mediators, ECs release serotonin [189], which activates the ENS and the extrinsic vagal afferents to the brain, and may also activate EGCs [21]. The involvement of serotonin has been demonstrated to play a key role in the regulation of intestinal secretion, gut motility, several GI disorders, nausea, vomiting, and acute gastroenteritis [190,191]. Rotaviruses can infect EC cells and stimulate serotonin secretion in a dose- and time-dependent manner, leading to RV-related diarrhea [192]. Similarly, Adenovirus-41 (HAdV-41) can stimulate serotonin from coxsackievirus and adenovirus receptor (CAR)-expressing human EC cells, activating EGCs. These observations highlight a serotonin-dependent cross talk between HAdV-41, EC cells, and EGCs that may be relevant for understanding how HAdV-41 causes diarrhea [193].

The activation of EGCs has been hypothesized for SARS-CoV-2 related-diarrhea. Indeed, the activated EGCs massively release IL-6 and other inflammatory mediators, resulting in the so-called “cytokine storm” observed in COVID-19 patients. Therefore, in these cases, GI dysfunction may be considered as a possible marker of involvement of ENS/EGC, rather than an accessory symptom, highlighting a pathophysiological mechanism underlying SARS-CoV-2 neuroinvasion [194,195,196,197].

Enteric neurons can be targeted by HSV-1. Once infected, the neurons recruit inflammatory macrophages that, by releasing ROS, induce changes in ENS neuroplasticity and trigger the destruction of the enteric ganglia, causing gastrointestinal dysmotility [198,199]. HSV-1 infection leads to the destruction of the enteric neurons by the massive recruitment of neutrophils, resulting in the permanent loss of peristalsis and the development of a toxic megacolon [172]. Therefore, the acute or chronic exposure of enteric neurons to neurotropic viruses, such as HSV-1, permanently disturbs the interplay between the ENS and immune cells.

The number of viruses physiologically residing in the human intestine is estimated to be up to 10^9^ per gram of feces [15], mainly comprising bacteriophages (prokaryotic-infecting viruses), and to a lesser extent, plant-, amoebae-, human-, and other animal-infecting viruses [200]. The human virome is mostly acquired postnatally and is influenced by a combination of dietary, maternal, and environmental sources [201]. During its life course, the virome diversifies and reaches its peak by adulthood [202]. Eukaryotic viruses, such as *Parvoviridae, Anelloviridae, Picobirnaviridae, Circoviridae,* and *Reoviridae*, are often part of the enteric virome of healthy humans [200], despite being opportunistic pathogens. It is not yet understood which viral sensing and signaling pathways are important for adjusting the immune responses to control the abundance and composition of the human intestinal virome.

## 5. Parasitic Influence on the Enteric Nervous System

The neuron-mediated response by the ENS and the immune system against a parasitic infection has not yet been fully elucidated [203]. The main findings in terms of the parasitical influence on the ENS are reported in Table 3 and represented graphically in Figure 2. Intestinal parasitic infections could unbalance the gut homeostasis, mainly through the modification of ENS components involved in neurotransmission, especially 5-HT production.

Intestinal parasites are capable of secreting low molecular weight substances, such as ammonia, urea, and amino acids, and high molecular weight protein molecules. Some substances secreted by parasites show great similarity to host neurotransmitters [204]. These substances indicate an attempt by the parasite to escape the host’s immune response and establish a favorable environment [205]. It is believed that some intestinal parasites (e.g., *Anisakis simplex* and *Schistosoma*) are able to produce and secrete acetylcholinesterase, which is an enzyme responsible for the degradation of acetylcholine, causing changes in the control of intestinal motility [206,207]. The presence of acetylcholinesterase in the intestine, even at extremely low concentrations, is capable of stimulating the growth and proliferation of intestinal mucosal epithelial cells [206,207]. Additionally, it has been shown that other neurotransmitters, such as GABA, serotonin, dopamine, and VIP, are present in intestinal parasites in both the larval and adult stages, ready to be secreted at the site of infection. For example, *Nippostrongylus brasiliensis* is able to produce and secrete a 30 kDa protein that has a great structural similarity to VIP. In experimental models, when the 30 kDa protein of parasitic origin was infused into the intestinal lumen, there was an intestinal reaction similar to that induced by VIP [208]. This protein was able, like VIP, to reduce the amplitude and frequency of contraction of the muscular layers of the intestine.

Other parasites, as in the case of *Cryptosporidium*, promote a VIPergic and cholinergic response via an increased expression of prostacyclin released by local neutrophils in response to *Cryptosporidium* infection [209,210]. Others, such as *Giardia duodenalis*, while reducing the number of 5-HT-secreting ECs, promote the selection of CCK-containing duodenal enterocyte via a mechanism that remains unknown [211,212,213]. CCK is involved in smooth muscle contraction and has a major effect on gall bladder contraction and the delivery of bile in the intestine, which is crucial for *Giardia* trophozoite growth. Furthermore, from what emerged from the muscle-myenteric plexus of *Trichinella spiralis*-infected rats, there is an increased expression of substance P of about 500% when compared to the levels found in the gastrointestinal tract not infected by parasites [214]. This neurotransmitter has an important function in the immune system, showing pro-inflammatory functions by improving lymphocyte and macrophage function. The interaction of substance P with its receptors directly induces vasodilation, which increases vascular permeability and allows the extravasation of plasma and degranulation of mast cells. The degranulation of mast cells releases histamine, which further amplifies vascular processes and activates nociceptors. Lymphocytes, granulocytes, and macrophages have receptors for substance P and these cells can be stimulated to produce cytokines. Macrophages stimulated by substance P produce the inflammatory mediators, prostaglandin E2 and thromboxane, as well as proinflammatory cytokines, IL-1, IL-6, and TNF. All of these molecular events support the synthesis and release of new molecules of substance P, thus perpetuating this vicious cycle [215,216].

Studies on experimental models have shown that in the face of an infection by intestinal parasites, the infusion of anti-substance P antibodies induces an immuno-neutralization picture in the gastrointestinal tract. These data indicate that the bioavailability of substance P represents an important component in the neurochemical response to intestinal inflammation induced by parasites [217]. Another component of the NANC enteric system that presents an important modification in the face of parasitic infection is intrinsic nitric oxide (iNO), which is identified in the enteric nervous system by the enzyme responsible for its synthesis—nitric oxide synthase (iNOS). This gas has a potent function in muscle relaxation and represents an important proinflammatory factor in the gastrointestinal tract. It has been shown that in the face of intestinal infection by parasites, iNOS levels are drastically reduced in the nerve plexuses, intestinal mucosa, and muscle layers [218]

At the same time, a conspicuous group of parasites may affect the ENS by promoting neuron destruction or phenotypic changes. Three examples are represented by *Entamoeba histolytica*, *Toxoplasma gondii*, and *Trypanosoma cruzi*.

*E. histolytica*, which is a widespread protozoan parasite that is endemic in developing countries and transmitted via the ingestion of infective cysts through contaminated food or water, was found to degrade ENS neurons, with a process involving cysteine proteases [219]. Moreover, when *E. histolytica*-secreted products or -soluble components were added to cell cultures, they decreased the neuron number by 30% and the axon number by 50%, with direct cytotoxicity being specific to the neuronal population [219].

*T. gondii* is an obligate, intracellular parasitic protozoan found worldwide, whose infection does not usually produce observable symptoms in healthy humans. *T. gondii* induces quantitative phenotypic changes in nitrergic neurons. These neurons, which at first do not release NO, begin secretion in response to INF-γ [220]. In addition, *T. gondii* induces a reduction of sub-mucosal VIP-reactive neurons and promotes the cytokine-induced death of enteroglial cells [221].

*T. cruzi* is a unicellular protozoan that is responsible for Chagas’ disease. In its chronic form, Chagas’ disease develops decades after the initial infection and alters the neuro-regulation of the heart and the entire gastrointestinal tract, especially the esophagus and colon. In the final stage of intestinal disease, the motility of the digestive system is impaired in such a way that it results in the dilation of intestinal segments (chagasic megacolon and megaesophagus). This form of the disease is accompanied by severe weight loss due to secondary achalasia of the lower esophageal sphincter. The chronic autonomic nervous pathology observed in Chagas’ disease has an autoimmune basis [222], with antibodies against neurons forming thanks to the cross-reactivity between the *T. cruzi* flagellar surface antigen and intra-axonal filaments [223]. In addition, *Trypanosoma amastigotes* can release a neurotoxin-like substance that has neurolytic properties [223].

Intestinal infection by parasites causes anatomical and functional changes in the muscular layers of the intestinal wall and in the ENS. It is possible to observe thickening of the external muscular layer of the small intestine of rats and guinea pigs and the colon of patients with the digestive form of Chagas’ disease [224], probably due to mediators directly originating from parasites or the inflammatory cascade. The increase in the thickness of the muscle layers is mainly due to the hypertrophy of the smooth longitudinal muscle layer (outer layer). The induction of hypertrophic changes in the intestinal smooth muscle in experimental models through the surgical induction of stenosis of a segment indicates that the “hypertrophy” of the intestinal musculature may actually represent an adaptive change, and studies indicate that it seems to be directly related to an increase in the neuronal and muscular response to serotonin expression [225].

Altered intestinal motility due to a modified contractility of the muscular layers and the consequent disturbance in fluid transport are the two constant pieces of evidence for parasitic infections [226]. However, the mechanisms involved vary between species. Some parasites will have a direct influence on the ENS, altering either the number of neurons or even the neuropeptide expression phenotype thereof. Other parasites, on the other hand, will influence the release of neurotransmitters, which, in turn, modify enteric activities and functions [227]. Currently, different studies using enteric parasites are also being carried out using strictly controlled clinical protocols as immunotherapeutic agents to articulate or restore the balance of the ENS in the face of human inflammatory bowel disease [228]. In addition, research on enteric helminths offers models for investigating the long-term consequences of enteric infections that produce functional bowel disorders, such as human irritable bowel syndrome, which occurs in the absence of inflammatory processes and without major histopathological changes [229]. The interaction between the parasite and host in the gastrointestinal tract will continue to provide effective experimental models that allow us to address vital issues to determine the integrative mechanisms involved in the neuroimmune modulation of the gastrointestinal tract function during infectious, allergic, and idiopathic states of intestinal diseases in mammalian hosts. In addition, the correlation between the host’s immune response, inflammatory mediators, and the enteric activity demonstrated in various parasitic infections is of equal importance to that given to the immune system, central nervous system, and gastrointestinal tract axis. Further research will investigate the role of parasite-induced microbiota in the intestinal neuroregulatory response during and after infection.

## 6. Conclusions

Accumulating evidence suggests that the development and homeostasis of the ENS are mediated by luminal microbial factors. In particular, pathogens may take advantage of ENS neurotransmitters to potentiate their action or even create an intestinal microenvironment suitable for their reproduction. In addition, the ENS may represent the first interface between the intestinal content and the CNS, thus explaining the intricate relationships behind intestinal microbes and their effects on CNS inflammation, behavior, and even actions. That being said, the current knowledge is still in its infancy and further studies are required. However, from the current knowledge emerges an interesting field that may shape the future concepts on pathogen–host synergic interactions. In our review, we have reported strong evidence to conceptualize future research.

## Figures and Tables

**Table 1:** Bacteria and the enteric nervous system.

Field of Interest	Key Findings
Gut Microbiota	
Social behavior	Social events allow horizontal transmission of microbes between individuals of the same species (as observed in Blattodea or baboons). Rodent models with high-fat diets and reduction of *Lactobacillus* spp. give birth to offspring with reduced ability to discriminate between familiar and unknown individuals of the same species. Dysbiosis promotes drastic changes in social behavior in rodents and supplementation with Bifidobacteria and Lactobacilli leads to improvement in early life and adulthood.
Sleep cycle and mood dysorders	Gut microbiota can alter sleep cycles through the systemic production of inflammatory cytokines, which have been proven to alter non-REM sleep and alter cortisol and norepinephrine production. These phenomena are related to gut permeability and systemic translocation of gut bacteria.
Alzheimer’s disease (AD)	Several bacteria promote neuro-inflammatory response typical of AD. Increased phosphorylated tau in patients with microbiota metabolites in cerebrospinal fluid.
Parkinson’s disease (PD)	High microbial density in the olfactory bulbs of patients with PD. Postural instability and gait symptoms can be associated with abundance of particular species.
**Pathogenic bacteria**	
Toxin-producing bacteria	Toxin-induced diarrhea is favored by the promotion of serotonin (5-HT) from the mucosa, resulting in activation of the secretomotor reflex pathways through local 5-HT receptors. In cases of emesis, 5-HT receptors are located in vagus nerve (VN) sensory terminals that project up to the emetic center in the brainstem.

Given the broad abundancy of information on the topic of interactions between bacteria (being pathogens or commensal bacteria), only key findings have been reported.

**Table 2:** Viruses and the enteric nervous system.

Viral Agent(s)	Pathogenetic Mechanism(s)	Disease(s)
TBEV	Myenteric plexus infection	Irreversible ileus
WNV, ZIKV	Viral replication within enteric neurons causing cell death	Intestinal dysmotility
Influenza A virus/HSV-1	Influenza A virus alterations in the ENS structures, followed by HSV-1 life-long persistency	Parkinson’s disease
Herpesviruses (EBV, VZV)	VZV latency in ganglia of the ENS; EBV induction of inflammatory infiltrates within the myenteric plexuses	Ogilvie’s syndrome, CIIPO, myenteric ganglionitis
JCV	Infection of the EGCs of the myenteric plexus	CIIPO
HIV	HIV-1 Tat protein activation of EGCs causing a neuroinflammatory response and synergistic action with morphine	Diarrhea and neurotoxic effects
Rotaviruses	Rotavirus infection of the EC cells and stimulation of serotonin secretion	Rotavirus-related diarrhea
HAdV-41	Serotonin release from EC cells leading to activation of EGCs	Diarrhea
HSV-1	Destruction of the enteric neurons by the massive recruitment of neutrophils	Loss of peristalsis and toxic megacolon
SARS-CoV-2	Activation of EGCs with massive release of IL-6 and other inflammatory mediators (cytokine storm)	SARS-CoV-2 related-diarrhea

The table reports the principal viral agents and their pathogenic mechanisms and the disease. TBE: Tick-Born Encephalitis; WNV: West-Nile Virus; ZIKV: Zika Virus; HSV-1: Herpes Simplex Virus-1; EBV: Epstein–Barr Virus; VZV: varicella zoster virus; JCV: John Cunningham Virus; EGCs: enteroglial cells; CIIPO: chronic intestinal idiopathic pseudo-obstruction; HIV: Human Immunodeficiency Virus; EC cells: enterochromaffin cells; HAdV-41: Adenovirus-41.

**Table 3:** Parasites and the enteric nervous system.

Parasite	Pathophysiological Modifications	Involved Factors
***Cryptosporidium*** ***parvum***	Altered transmembrane ionic transport/hypersecretion	Cholinergic and VIPergic response through prostacyclins Increased levels of substance P
***Giardia duodenalis***	Altered intestinal contractility Promotion of malabsorption and hypersecretion	Depletion in NO synthesis Reduction of 5-HT secretion Increase of CCK secretion
***Entamoeba histolytica***	Neuron and axon depletion	Process dependent on cysteine-proteases
***Nippostrongylus brasiliensis***	Motility dysfunction	Production of a VIP-similar peptide
***Trichinella spiralis***	Increased intestinal contractility	Altered neurotransmitter releases with 5-HT receptor disfunction
***Trypanosoma cruzi***	Decrease of enteric glial cells	Cross reaction between parasitic antigens and the human hosts Reduced contractility due to loss of Ach receptor function
***Toxoplasma gondii***	Phenotypic changes in enteric neurons	Increased NO response Reduction of VIPergic neurons

NO: nitric oxide; 5-HT: 5-hydroxytryptamine; CCK: cholecystokinin; Ach: acetylcholine.
